# Testosterone deficiency in non-cancer opioid-treated patients

**DOI:** 10.1007/s40618-018-0964-3

**Published:** 2018-10-20

**Authors:** F. Coluzzi, D. Billeci, M. Maggi, G. Corona

**Affiliations:** 1 Department of Medical and Surgical Sciences and Biotechnologies, Sapienza University of Rome, Latina, Italy; 2 Neurosurgical Department, ULSS2 Treviso Hospital, University of Padua, Treviso, Italy; 3 Department of Experimental and Clinical Biomedical Sciences, Sexual Medicine and Andrology Unit, University of Florence, Florence, Italy; 4 Endocrinology Unit, Medical Department, Azienda Usl di Bologna, Maggiore-Bellaria Hospital, Largo Nigrisoli 2, 40133 Bologna, Italy

**Keywords:** Opioids, Chronic non-cancer pain, Opioid-induced androgen deficiency, Testosterone

## Abstract

**Purpose:**

The use of opioids in patients with chronic non-cancer pain is common and can be associated with opioid-induced androgen deficiency (OPIAD) in men. This review aims to evaluate the current literature regarding the prevalence, clinical consequence and management of OPIAD.

**Methods:**

A database search was performed in Medline, Embase and Cochrane using terms such as “analgesics”, “opioids” and “testosterone”. Relevant literature from January 1969 to March 2018 was evaluated.

**Results:**

The prevalence of patients with OPIAD ranges from 19 to 86%, depending on the criteria for diagnosis of hypogonadism. The opioid-induced suppression of gonadotropin-releasing and luteinizing hormones represents the main important pathogenetic mechanisms. OPIAD has significant negative clinical consequences on sexual function, mood, bone density and body composition. In addition, OPIAD can also impair pain control leading to hyperalgesia, which can contribute to sexual dysfunction and mood impairment.

**Conclusions:**

OPIAD is a common adverse effect of opioid treatment and contributes to sexual dysfunction, impairs pain relief and reduces overall quality of life. The evaluation of serum testosterone levels should be considered in male chronic opioid users and the decision to initiate testosterone treatment should be based on the clinical profile of individuals, in consultation with the patient.

## Introduction

Chronic non-cancer pain (CNCP) is common worldwide, with an estimated prevalence of 8–60% [1, 2], and profoundly impacts the overall quality of life and mental health of affected individuals [1, 2]. CNCP is a significant economic burden for patients, health services and societies, costing an estimated $500 billion per annum in the USA, due to the impact on absenteeism and worker productivity [3]. Moreover, the burden of CNCP is expected to increase as a consequence of an aging population and increasing levels of obesity, due to lack of physical activity and urbanization [3].

Opioids are an integral part of the World Health Organization (WHO) analgesic ladder for cancer pain. In the last decade, opioids have also been widely used for CNCP; however, the epidemic of opioid overdose, recently recorded in the USA, has led to concerns about their safety [4]. Weak opioids or low doses of strong opioids may be safer than non-steroidal anti-inflammatory drugs (NSAIDs), particularly in the elderly population, as opioids do not impair organ function [5]. However, the effectiveness of opioids for long-term use in CNCP has not been established and the risk of opioid-related abuse or overdose should be avoided. Accordingly, recent guidelines from the US Centers for Disease Control and Prevention (CDC) recognize that non-opioid therapy is preferred for the treatment of CNCP [6]. Opioids should be used only when the benefits for pain and function outweigh risks [6]; this is supported by other evidence in the literature [7–9].

Adverse events (AEs) are common in patients treated with opioids and usually begin soon after the initiation of treatment. The most common AEs occur in the gastrointestinal tract and central nervous system (CNS), but new concerns are emerging about potential effects on bone and joint physiology [10]. Although patients develop tolerance for nausea, vomiting and CNS AEs, this is not the case for bowel dysfunction, which can persist during the entire course of treatment [11, 12].

**Fig. 1:**
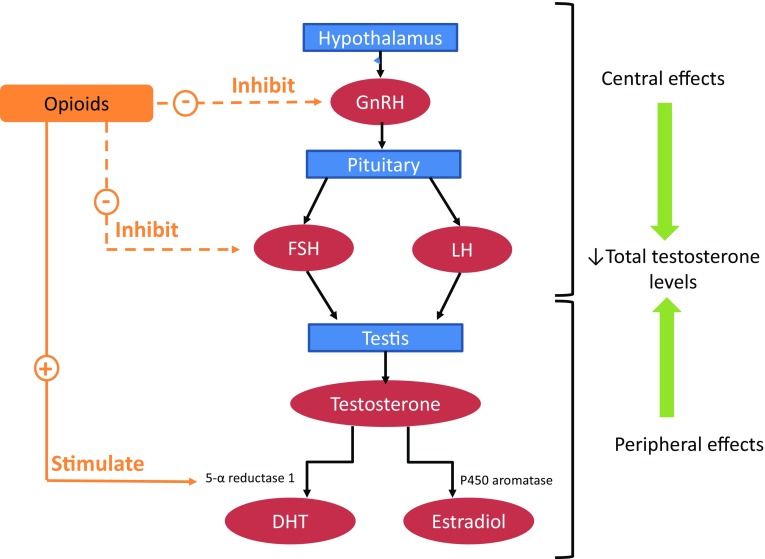
Summary of mechanisms involved in the pathogenesis of opioid-induced androgen deficiency. *GnRH* gonadotropin-releasing hormone, *FSH* follicular-stimulating hormone, *LH* luteinizing hormone, *DHT* dihydrotestosterone

The endocrine system can also be severely affected by chronic opioid treatment. Opioids can influence the secretion of hormones at different levels of the hypothalamus–pituitary–gonadal (HPG) axis [13]. Opioids generally increase the levels of growth hormone, thyroid-stimulating hormone and prolactin, but there are conflicting reports on the effects of opioids on arginine vasopressin and adrenocorticotropic hormone [13]. In addition, opioids can lead to the development of hypogonadism by directly inhibiting gonadotropin-releasing hormone (GnRH) through the µ-opioid (MOP) receptor (Fig. 1), reducing libido and causing erectile dysfunction (ED) in men, oligomenorrhea or amenorrhea in women, and bone loss or infertility in both sexes [13]. The clinical significance of opioid-induced androgen deficiency (OPIAD) and the contribution of testosterone treatment (TTh) to improve symptoms of OPIAD has not been completely clarified [14–18]. Furthermore, the role of TTh in medical conditions other than classical hypogonadism has been recently the subject of discussion [19]. The aim of this review is to evaluate the data on the prevalence of OPIAD and the risk/benefit ratio of TTh in this condition.

## Methods

A comprehensive Medline, Embase and Cochrane search was performed including the following words: (“analgesics, opioid”[Pharmacological Action] OR “analgesics, opioid”[MeSH Terms] OR (“analgesics”[All Fields] AND “opioid”[All Fields]) OR “opioid analgesics”[All Fields] OR “opioid”[All Fields]) AND (“testosterone”[MeSH Terms] OR “testosterone”[All Fields]).

Publications from January 1, 1969 up to March 30, 2018 were included.

### Opioids and their use in CNCP

Opioids exert their analgesic effects in humans via agonist, partial agonist or antagonist activity on opioid receptors, δ (DOP), κ (KOP) and μ (MOP) [20]. The classical G-protein-coupled opioid receptors, known as DOP, KOP and MOP [20], are widely distributed in the CNS and to a lesser extent in the periphery [21]. Various endogenous ligands derived from pro-hormone precursors act at the receptors. Proenkephalin binds to DOP receptors and is cleaved to form met-enkephalin and leu-enkephalin [22]. Dynorphin A and B, derived from prodynorphin, are agonists at the KOP receptor [23]. Proopiomelanocortin is the precursor of β–endorphin, which has agonist activity in all three opioid receptors, but principally the MOP receptor [24]. The nociceptin (NOP) receptor is a ‘non-classical’ G-protein-coupled receptor; its endogenous agonist is derived from a precursor compound, pre-pronociceptin. The NOP receptor is insensitive to the prototypical opioid agonist morphine and antagonist naloxone (Table 1).

**Table 1:** Opioid receptors with their endogenous ligands, precursors and clinical drugs with agonist or antagonist activity

	Receptor subtype			
	DOP (δ)	KOP (κ)	MOP (μ)	NOP
Precursor	Proenkephalin	Prodynorphin	Proopiomelancortin	Pre-pronociceptin
Peptide	Met-enkephalin, leu-enkephalin	Dynorphin A, dynorphin B	β–endorphin	N/OFQ
Agonists				
Morphine	+	+	+++	−
Fentanyl	−	+	+++	−
Codeine	++	++	++	−
Oxycodone	−	+++	+++	−
Buprenorphine	−	+	++	+
Tramadol	−	−	+	−
Methadone	+	−	++	−
Meperidine	+	+	++	−
Tapentadol	−	+	+	−
Antagonist				
Naloxone	++	++	+++	−

− No affinity; + low affinity; ++ intermediate affinity, +++ high affinity

*N/OFQ* nociception orphanin FQ, *DOP* delta opioid receptor, *KOP* kappa opioid receptor, *MOP* mu opioid receptor, *NOP* nociceptin opioid receptor

Opioids have been widely used for CNCP, with specific recommendations about patient selection, treatment tailoring, dose titration, maintenance therapy, and tapering doses during discontinuation [25]. Despite some critical points, one in five US patients with CNCP receive prescription opioids [26]. In particular, their endocrine effects can be minimized by using drugs with a lower MOP affinity.

Opioids for CNCP are commonly classified, according to their analgesic potency, as weak (codeine and tramadol) for mild-to-moderate chronic pain, or strong opioids (morphine, oxycodone, fentanyl, hydromorphone, methadone, buprenorphine, tapentadol) for severe chronic pain. They may be also classified according to their duration of action: long-acting opioids (LAO), used to maintain a stable plasma dose during chronic therapy, short-acting opioids (SAO), mainly used for titration at the beginning of opioid therapy, and rapid-onset opioids (ROO) indicated for breakthrough cancer pain.

Codeine is a pro-drug that requires metabolic conversion to morphine for analgesic activity. A small proportion of the population (5–10%) lacks the enzymes for this metabolic activation and as such are unable to derive pain relief from codeine (poor metabolizers) [27]. It is available in fixed combination with paracetamol (acetaminophen) for mild-to-moderate pain [28].

Tramadol is a synthetic piperidine analog of morphine. It acts as a central analgesic via MOP receptors and by modulating monoaminergic (serotonin and noradrenaline) pathways. Because its activity on the MOP receptor is significantly lower than that of morphine [29], tramadol has less potential for respiratory depression and gastrointestinal side effects [30, 31]. Tramadol is available in different formulations as SAO, LAO, and in association with paracetamol for mild-to-moderate chronic pain [32, 33].

Another weak opioid is meperidine. This agent has approximately 10% of the efficacy of morphine and a half-life of only 3 h after oral administration [34]. It is rapidly metabolized to normeperidine, which has a half-life of 8–12 h and is associated with significant CNS AEs [34].

The strong opioid morphine has been the mainstay of chronic pain management. Morphine acts as a full agonist of the MOP receptor, with weak agonist activity at DOP and KOP receptors (Table 1) [35]. It is commonly administered via the oral, intravenous, or intrathecal routes, but morphine has low lipid solubility, resulting in slow penetration through the blood–brain barrier [36]. Therefore, the onset of the analgesic effect is relatively slow and the relative hydrophilicity impairs its administration through the skin. Oral bioavailability is about 30%, because morphine undergoes extensive hepatic first-pass metabolism, via glucuronidation [37]. Morphine has been shown to significantly decrease testosterone plasma levels, probably due to the action on opioid receptors present in the testis [17].

Oxycodone is a semi-synthetic derivative of thebaine with high oral bioavailability [38]. The extended-release formulations provide stable plasma levels throughout the dosing interval and allow twice-daily administration [39]. The fixed combination of oxycodone–naloxone 2:1 has significantly improved the gastrointestinal tolerability of oxycodone, reducing the incidence of opioid-induced bowel dysfunction [40]. However, when administered in males, oxycodone significantly reduces testosterone levels; in one study, 45.5% of patients treated with oxycodone (mean 75 mg/day) had testosterone values below the normal reference range after 12 weeks of treatment [41].

Hydromorphone is available for chronic pain management as a once-daily formulation, using an osmotic-controlled-release oral delivery system (OROS^®^). This formulation is minimally affected by food or alcohol, provides stable plasma concentrations and enhances patient adherence to treatment by reducing dosing frequency [42].

Fentanyl is a synthetic opioid analgesic with high lipid solubility and a faster onset of action than morphine [43]. All ROOs are fentanyl based, but their use is not indicated for CNCP. In chronic pain management, fentanyl is mainly used in transdermal formulations. Compared with oral opioids, transdermal fentanyl is associated with a lower incidence of side effects (constipation, nausea and vomiting, and daytime drowsiness), increased patient satisfaction and quality of life, and improved compliance resulting from administration every 72 h [44]. However, the convenience of the fentanyl patch should be weighed against the highest risk of androgen deficiency, when compared with other commonly prescribed opioids [45].

The “atypical opioid” tapentadol has a pharmacological profile that is distinct from all other opioids. It has a dual mechanism of action: µ-opioid receptor agonist/noradrenaline reuptake inhibitor (MOR/NRI). The affinity of tapentadol for the MOP receptor is 50 times lower than that of morphine, which, when counterbalanced by its activity on noradrenaline modulation, results in an improved tolerability profile, lower rate of tolerance development, lower abuse potential, and efficacy in both nociceptive and neuropathic pain [46, 47]. Tapentadol has been mainly studied in CNCP, including low back pain (LBP), neck pain, osteoarthritis, and painful diabetic peripheral neuropathy [48–50]. Compared with oxycodone/naloxone, tapentadol at the mean dosage of about 378 mg/day had minimal or no significant effects on testosterone levels in male patients with severe chronic LBP [41], probably related to the dual mechanism of action [51].

Buprenorphine also differs from traditional opioids (pure MOP agonists), because it works as a non-selective partial opioid agonist. It produces analgesic effects via MOP receptors and also antagonizes morphine antinociception at high doses via interaction with the KOP and NOP receptors [52]. Furthermore, compared with pure MOP agonists, buprenorphine has reduced potential for respiratory depression, overdose, and abuse, making it a useful drug for patients with chronic pain. At low dosages, transdermal buprenorphine is an accepted around-the-clock pain reliever for mild-to-moderate chronic pain [53]. Unlike morphine, buprenorphine has only limited endocrine effects and can be used for months without inducing hypogonadism [17].

Methadone is a synthetically derived opioid with agonist affinity for both the MOP and DOP receptors. Methadone has improved safety and tolerability compared with morphine because of its lower affinity for the MOP receptor. Methadone is mainly used in patients with chronic cancer pain who have reached a high opioid dose or in patients with a history of substance abuse as part of a methadone maintenance program (MMT) [54]. In these patients using high-dose MMT, low serum testosterone levels have been reported: the percentage of hypogonadism in men reached 75%, while in women was only 21% [55].

### Opioid-induced androgen deficiency

#### Prevalence

The association between reduced testosterone levels, androgen deficiency and opioid treatment has been documented since the 1970s, when reports emerged in men who were on maintenance methadone therapy [56, 57]. Since then, several studies with a limited number of patients have also suggested an association between testosterone suppression and opioid treatment [16–18]. A meta-analysis performed in 2015, including 17 studies and 800 opioid users and 1969 controls, concluded that mean testosterone levels were significantly lower in men treated with opioids compared with controls [58]. However, the analysis included a heterogeneous group of studies in men treated with opioids for different reasons. This represents an important limitation because comorbidities can profoundly influence testosterone levels. Addictive disorders, such as alcohol or opioid dependence, are frequently associated with human immunodeficiency virus (HIV) and chronic hepatitis, which can affect testosterone metabolism through direct (hepatic impairment) and indirect (use of retroviral drugs) mechanisms [59–61].

Studies evaluating the impact of opioid treatment on testosterone levels in men with CNCP show that the prevalence of OPIAD ranges from 19 to 86%, depending on the testosterone threshold used to define hypogonadism (Table 2) [45, 55, 62–72]. While OPIAD definitions vary, most studies report an overall prevalence higher than 50%, confirming the significant impact of opioids in reducing testosterone levels. Although few studies have investigated the impact of opioid dosage or specific drug characteristics on testosterone levels, the available evidence suggests an increased risk of androgen deficiency with higher opioid doses and with SAO than LAO formulations. However, in equivalent doses, LAOs are significantly more likely than SAOs to cause androgen deficiency in men [69]. Doses exceeding approximately 100 mg of oral morphine equivalent may be more likely to trigger the onset of OPIAD, compared with lower opioid doses [73], although this finding has not been confirmed by other researchers [56, 65]. In a more recent study comparing different opioid formulations, neither the chemical structure nor the lipophilicity of the opioid explained the androgen suppression [45]. Certain opioids are associated with an increased risk of androgen deficiency. In particular, the risk of androgen deficiency is highest with fentanyl, followed by methadone and oxycodone [45]. Tapentadol, which has a 50-fold lower affinity for the human MOP receptor than morphine, has a lower impact on sex hormone concentrations, compared with pure opioid analgesics, supporting the role of MOP receptors in the pathogenesis of OPIAD [41, 74]. In randomized, comparative studies, morphine and controlled-release oxycodone reduced serum testosterone levels to a greater extent than extended-release tapentadol [41, 74]. The presence of comorbidities, particularly diabetes, hypertension and dyslipidemia, further increases the risk of OPIAD [45]. This is not surprising as several studies have documented the influence of metabolic diseases on different levels of the hypothalamus–pituitary–testis axis and testosterone production [75–78].

**Table 2:** Summary of the literature evaluating the impact of opioid treatment on testosterone levels in men with non-cancer pain

Publication	Study design	*N*	Age (years)	Type of opioid/route of administration	MSE (mg)	Mean testosterone level during opioids (nmol/L)	Testosterone threshold for defining hypogonadism (nmol/L)	Proportion with hypogonadism (%)
Abs et al. [62]	Cohort	29	48.4	Morphine/intrathecal	4.8	6.8	9.0	86.2
Finch et al. [63]	Cross-sectional	11	46.5	Morphine/intrathecal	0.5–40	4.8	10.0	100
Daniell et al. [64]	Cross-sectional	23	49.4	Mixed/oral	70–120	6.5	NR	74.0
Roberts et al. [65]	Cohort	10	52.4	Mixed/oral or intrathecal	3.3	3.9	10.0	100
Rajagopal et al. [66]	Cross-sectional	20	50.1	Mixed/oral	> 200	3.9	8.3	90
Fraser et al. [55]	Cross-sectional	12	45.4	Mixed/oral or transdermal	718	6.9	Age specific range	83
Duarte et al. [67]	Cross-sectional	20	58.0	Morphine/intrathecal	2.68	4.9	8	85
Rubinstein et al. [68]	Cross-sectional	81	26–79	Mixed/oral	184	NR	8.6	53
Kim et al. [72]	Cross-sectional	8	60.1	Intrathecal	12.3	9.1	NR	50
Rubinstein et al. [69]	Cross-sectional	1585	54.0	Mixed/oral	76	NR	8.6	43.6
Cepeda et al. [71]	Cross-sectional	146	49.1	Mixed/oral	NR	11.8	10	35.1
Ajo et al. [70]	Cross-sectional	120	58.4	Mixed/oral	77	NR	10	19
Rubinstein et al. [45]	Cross-sectional	1159	53.1	Mixed/oral	44	9.9	8.6	38.9

*MSE* morphine sulfate equivalent, *NR* not reported

#### Pathophysiology

##### Animal studies

The primary mechanism by which opioids affect testosterone production is by inhibiting the secretion of GnRH (Fig. 1) [12]. Injections of β-endorphin in the ventromedial, anterior and preoptic-septal hypothalamic areas decrease the secretion of luteinizing hormone (LH) from the pituitary [79]. Li and Pelletier [80] used in situ hybridization to demonstrate that morphine down-regulates GnRH mRNA levels. Accordingly, chronic morphine administration inhibits GnRH secretion [13]. However, follicle-stimulating hormone (FSH) levels are not affected by opioid analogs or antagonists [13]. Animal models have also shown that opioids can modulate the negative feedback of sex steroids on LH secretion [13]. In male rats, morphine enhances the sensitivity of the hypothalamus to negative feedback by testosterone [81]. Lastly, there is a possibility that opioids can also influence peripheral testosterone metabolism. In a rat model, morphine administration increased the expression of 5-α reductase type 1 and/or P450-aromatase mRNA in different body regions (Fig. 1) [82].

##### Human studies

The available data on the OPIAD mechanism in humans reflects the findings of animal models (Fig. 1) [13]. In healthy, adult men, morphine decreases LH pulse frequency, but does not affect FSH levels [13]. It was suggested that treatment with opioids impairs gonadotropin pulse amplitude or affects the response of the anterior pituitary to GnRH, leading to a decrease in testosterone levels [83]. Interestingly, the effect of opioids on the human hypothalamus–pituitary–testis axis differs according to pubertal stage [13]. For example, naloxone administration failed to increase LH levels in early puberty, whereas it had a stimulatory effect in late pubertal boys [84], suggesting that regulation of central opioid receptors by sex hormones is dependent on sexual maturity [13]. In addition, opioid tone at the hypothalamic–pituitary level seems to change in an age-dependent manner. Indeed, in healthy elderly men, opioid tone was lower than in young men, as demonstrated by a reduction in the LH pulse amplitude and the frequency of high-amplitude pulses after naltrexone administration [85].

#### Clinical consequences

Opioids can induce several hypogonadism-related signs and symptoms, including sexual dysfunction, mood impairment, fatigue, weight gain and osteoporosis [16–18, 86], and these are described in more detail in the following sections. Regardless of the origin of hypogonadism, signs and symptoms are dependent on the age at onset [87, 88]. In adults and aging men, sexual dysfunction is the most specific symptom associated with low testosterone [89–91]. The European Male Aging Study, a population-based survey of more than 3400 men across eight European centers, showed that low libido, reduced spontaneity and sex-related ED were specifically associated with decreased testosterone levels, whereas other hypogonadal symptoms (e.g., physical and psychological changes) were less specific [89]. Similar results have been more recently reported in a population of men consulting for sexual dysfunction [100]. Furthermore, a large body of evidence has demonstrated that, although sexual dysfunction is the most specific symptom of adult-onset hypogonadism, central obesity may be both a cause and effect of hypogonadism [92, 93].

As well as opioids affecting testosterone levels, testosterone may also be involved in the regulation of endogenous opioid activity. In fact, androgen receptors are involved in the transcriptional regulation of the MOP receptor [94]. Therefore, in men treated with opioids, hypogonadism may manifest as poor control of pain and hyperalgesia, which can in turn cause sexual dysfunction and mood impairment.

##### Sexual function

Testosterone regulates male sexual function at both a central and peripheral level [95], so the association between sexual dysfunction and OPIAD is not surprising. However, it is important to recognize that chronic pain can also negatively affect sexual function through its associated disabilities, physical limitations and psychological distress [96, 97]. In addition, opioids can modulate sexual behavior by acting on the hypothalamic nuclei in the medial preoptic area and the spinal cord [13]. Further evidence supporting the negative impact of the endogenous opioid system on sexual function is derived from data obtained in patients with non-organic ED. Jannini and his group, in Italy, produced compelling evidence that robustly substantiated the hypothesis of an LH-mediated, sex-induced, drive in testosterone production [98–100] They essentially found that restoring sexual activity in patients affected by ED led to recovery of an otherwise borderline low testosterone level. The testosterone level rise was independent of the type of ED therapy employed, but was closely related to the successful outcome of therapeutic intervention. Hence, they speculated that sexual inertia resets the reproductive axis to a lower level of activity, somehow inducing a secondary hypogonadism, characterized by reduced LH bioactivity [100]. Consequently, restoring sex restores sex hormones, including bioavailable LH and testosterone [98–100]. The increased tone of the endogenous opioid system is the working hypothesis used by these researchers to explain their findings [101, 102]. Accordingly, the same researchers reported that the administration of naltrexone therapy for 7–15 days significantly increased the rate of successful coitus compared with placebo in these men [103].

Despite this evidence, few studies using standardized methods have evaluated the presence of sexual dysfunction in men receiving long-term opioids. In addition, most studies evaluating the effect of opioids on sexual function have been conducted in people with addictive disorders, limiting the generalizability of their findings [13]. A large cross-sectional study in 11,327 men with back pain found that the long-term use of opioids was associated with increased use of phosphodiesterase type 5 inhibitors or TTh [104]. In addition, patients prescribed a daily opioid dosage of at least 120 mg morphine equivalents reported higher use of medication for ED compared with those receiving lower opioid doses [104]. A study of 120 men with CNCP, who had been receiving opioids for at least 12 months, found that 78% reported sexual dysfunction according to the International Index of Erectile Function (IIEF) score [70]. In most of these patients (69%), the sexual dysfunction was severe [96]. In this study, 19% of patients had OPIAD, defined as a total testosterone level < 10.4 nmol/L, and IIEF scores were significantly lower in those with OPIAD than in eugonadal men [70]. IIEF scores were worse in patients receiving oxycodone than other opioids [70], consistent with its more marked effect in reducing testosterone levels [45]. In other comparative studies of OPIAD and sexual dysfunction, buprenorphine (a partial μ-opioid agonist used for opioid dependence) caused less marked reduction of testosterone levels and a lower incidence of sexual dysfunction, compared with methadone [105]. Standards have not been established for monitoring and treating OPIAD patients. However, according to the literature and expert opinion, patients who use opioids at doses higher than 100 mg of morphine equivalents per day should be monitored for the development of hypogonadism [86].

##### Mood

The association between CNCP, depression and reduced quality of life is well documented [106]. In a large national sample of patients prescribed opioids for CNCP in Australia (*n* = 1418), 61% had ever experienced depression, and 66% of those patients reported depressive symptoms in the previous 12 months [107]. Among those who had reported lifetime depression, 48% developed depression after the onset of pain and the initiation of opioid medications. Several reports have also documented a dose- and duration-dependent association between chronic opioid treatment and new-onset depression [106]. One possible reason for this association is a reduction in testosterone levels caused by opioid administration [108, 109], with OPIAD contributing to the development of depression in patients with CNCP. However, the relationship between low testosterone levels and the incidence of clinical depression is still unclear, as is the potential benefit of TTh in men with depression or OPIAD [109].

##### Bone

Osteopenia and osteoporosis are well-known consequences of male hypogonadism [110]. Osteopenia has been reported in 50% of men treated with opioids for CNCP [55], and epidemiological studies have shown that opioid treatment is associated with a 50–60% increase in the risk of osteoporotic fractures [86, 111]. Patients older than 60 years, who use opioids equivalent to 50 mg/day or higher, have a 10% fracture rate per year, with a twofold higher risk than patients not using opioids [112]. Besides OPIAD, other factors may explain the relationship between opioid use and osteoporotic fractures. Opioids have a direct effect on bone formation by impairing osteoblastic activity via MOP receptors [86, 111]. In animal models, tramadol, which has lower affinity for the MOP receptor than morphine or fentanyl, is also associated with a lower incidence of osteoporosis than these agents [13]. The incidence of fractures may also be affected by the CNS effects of opioids, such as dizziness and sedation, which can play an important role in opioid-related falls [86].

##### Body composition and cardiovascular risk

Several epidemiological studies have documented an association between CNCP, opioid use and obesity [113, 114]. The reasons of this association are multifactorial. Physical limitations and depression related to CNCP may reduce energy expenditure and increase food intake [113, 114]. Conversely, opioids can modulate eating behavior [115]; as such, the combination therapy of naltrexone and bupropion has been approved for the treatment of obesity [115]. In addition, OPIAD can contribute to opioid-induced obesity. Observational studies have documented a close association between hypogonadism in adulthood and obesity or metabolic diseases [78, 92], partially restored by TTh [76, 116–118].

A possible relationship between low testosterone and increased cardiovascular risk has also been suggested [119–121]. However, it is not clear whether low testosterone in aging men plays a direct pathogenic role in cardiovascular disease, is a marker of poor health, or even a protective mechanism, turning off testosterone-dependent functions (such as reproduction and/or physical and sexual activity) when cardiovascular function is impaired [122, 123]. A large UK general practice research database found an increased risk of myocardial infarction in 1.7 million patients with at least one prescription for an opioid to treat CNCP between 1990 and 2008, especially in patients treated with morphine, meperidine and polytherapy [124]. The possible contribution of OPIAD was not investigated in this study.

#### Management of opioid-induced androgen deficiency

OPIAD is a reversible condition, and opioid withdrawal may normalize testosterone levels within 1 month in subjects with heroin addiction [125]. Similar data are not available in men with CNCP, but discontinuing an opioid molecule with lower effect on the HPG axis may be an option for symptomatic men with reduced testosterone levels. If discontinuing opioids is mandatory, non-opioid treatment should be considered for managing chronic pain in these patients. According to current guidelines, if pain relief with non-opioids is inadequate or patients are unable to discontinue opioid treatment, TTh should be considered [126–128]. A limited number of studies have evaluated the effects of TTh in men with OPIAD (Table 3) [129–133]. In the only randomized controlled trial available, TTh reduced mechanical hyperalgesia and improved sexual desire and overall quality of life [130]. The improvement of sexual function and pain relief after TTh was confirmed in other prospective and retrospective observational trials [129, 131–133]. The effects of TTh observed in men with OPIAD are consistent with the effects observed in the general population [109, 119, 134]. There is no general agreement among different societies on the ideal minimum levels of total testosterone for initiating TTh; the International Society for Sexual Medicine and the Endocrine Society have defined a minimum testosterone level of 12 nmol/L and 9.4 nmol/L, respectively, [126, 128]. Meta-analyses have shown that TTh improves all aspects of sexual function when the total testosterone level is below 12 nmol/L (3.5 ng/mL) [134, 135]. Similar beneficial effects of TTh have been observed for other outcomes, such as body composition (central adiposity, lean body mass) and glycometabolic parameters [109, 119].

**Table 3:** Summary of the studies evaluating the effects of testosterone treatment in men with opioid-induced androgen deficiency

Publication	Study design	*N*	Age (years)	Testosterone (nmol/L)	Study duration (weeks)	Comparator	Sexual function	Pain	Mood	QoL	Body composition
Daniell et al. [129]	Observational prospective	23	46.0	6.9	24	NA	↑	↑	↑	↑	NR
Aloisi et al. [131]	Observational prospective	17	61.3	4	52	NA	↑	↑	↔	↑	NR
Blick et al. [132]	Observational prospective*	90	48.3	9.8	52	759 NOU	↑	↑	↑	NR	↔
Basaria et al. [130]	RCT placebo controlled	43	48.0	8.3	14	41	↑	↑	NR	NR	↑
Raheem et al. [133]	Observational retrospective	11	55.0	8.1	78	16 men with untreated OPIAD	↑	↑	NR	NR	NR

*Data are from the Testim Registry in the USA

↑ Increased/improved; ↔ unchanged

*NA* not applicable, *NOU* non-opioid users, *NR* not reported, *OPIAD* opioid-induced androgen deficiency, *QoL* quality of life, *RCT* randomized controlled trial

The effects of available testosterone preparations have been previously reviewed [109, 136]. In summary, transdermal gel preparations, as well as long-acting injectable testosterone undecanoate, should be preferred over older short-acting injectable or oral testosterone formulations due to their better efficacy and safety profiles [109, 136, 137]. Side-by-side comparisons of different testosterone products are lacking and the choice of testosterone preparation should be discussed with the patient and the final decision based on the clinical situation.

## Conclusions

OPIAD is a common AE of opioid treatment in men with CNCP. The presence of OPIAD can negatively affect pain relief and impair sexual function, mood and overall quality of life. However, it is important to recognize that testosterone levels in men progressively decrease as a function of age and comorbidities, which are common in patients with CNCP. Therefore, physicians should consider measuring testosterone levels in all men, before prescribing opioid treatment, and selecting opioids with a lower MOP affinity. In symptomatic patients with OPIAD, if opioid discontinuation is unlikely, TTh may be initiated; however, this decision should be discussed with the patient, emphasizing that, although there is some evidence to support positive short-term outcomes, the long-term effect of TTh in men with OPIAD is unknown.
